# The "Full-Time" Teacher

**Published:** 1920-05-22

**Authors:** 


### THE "FULL-TIME" TEACHER.

An American medical contemporary tells us with more than a touch of the enthusiasm typical of that very live country that in their medical schools "full-time men are becoming all the rage," and that, in practically all those institutions which can afford it, even the younger members of their staffs are being placed in such a position that their interests are not divided by the calls of private practice. Unfortunately, we cannot at the moment judge if this description accurately represents the immediate trans-Atlantic situation or not, but it appears, from the assurance and the authority which the author apparently possesses, that in America appreciation and development of the full-time teaching unit is being shown far more actively and widely than it is in our own country, in spite of the few recent instances of academic reconstruction which we have been able to report. Whether this extreme speeding up of reform will fulfil all the brightest hopes of the optimist remains to be seen, for, in any case, it seems probable that, with the now closer professional cooperation between the two countries, the activities of the Fellowship of Medicine and similar associations, the final result will be much the same in both. Yet there are certain considerations to be made before allowing our younger hospital teachers completely to turn their backs upon treatment of disease outside the institutional walls.

It is true that such men become relatively free agents who can spend all their time in the pursuit of true science, and therefore, in an academic sense, they present to the students medicine at its best, and as seen through the eyes of the true investigator. Also we must admit that the arrangement which thus develops a teacher gradually is better than the method of appointing the man with the largest reputation, and perhaps largest practice, to hurry in his free moments to tutorial duties at a medical school. But is it advisable completely to extinguish the practitioner-teacher as the extremists would wish us to do?

However completely a matured teacher, whose own experience has been of hospital practice alone, may realise the limitations and demands of medicine as carried on in the private homes of the people, he can never really convey to his students just that point of view which it is absolutely essential for the good general practitioner to hold. In any circumstances let us remember that an army of really well equipped general workers is a national necessity in the future, however much our appreciation of and enthusiasm for research is meanwhile increased. Therefore we are convinced that, although some members of the teaching staff should, as with the new "units" in England, be advisedly full-time men and their own training developed true to type, yet it would be particularly detrimental to medicine of the future if the old-time methods of clinical teaching were done away with completely. In short we must have *both* types of teachers and therefore always have *both* types with us in the making. The full-time man is an invaluable adjunct to our medical schools' resources, but to change over to an entirely full-time staff would be passing from the old to another and an undoubtedly worse extreme.

